# An unclear partnership: key questions about physician and advanced practice provider collaboration in primary care

**DOI:** 10.1093/haschl/qxaf006

**Published:** 2025-01-17

**Authors:** Estelle Martin, Bruce Landon, Joanne Spetz, Susan Edgman-Levitan, Hannah Neprash, David W Bates, Lisa Rotenstein

**Affiliations:** Division of Clinical Informatics and Digital Transformation, University of California at San Francisco, San Francisco, CA 94143, United States; Center for Physician Experience and Practice Excellence, Brigham and Women's Hospital, Boston, MA 02115, United States; University of California at San Francisco, San Francisco, CA 94143, United States; Division of General Internal Medicine, Beth Israel Deaconess Medical Center, Boston, MA 02215, United States; Department of Healthcare Policy, Harvard Medical School, Boston, MA 02115, United States; Philip R. Lee Institute for Health Policy Studies, University of California at San Francisco, San Francisco, CA 94158, United States; Stoeckle Center for Primary Care Innovation, Massachusetts General Hospital, Boston, MA 02114, United States; Division of Health Policy and Management, School of Public Health, University of Minnesota, Minneapolis, MN 55455, United States; Center for Physician Experience and Practice Excellence, Brigham and Women's Hospital, Boston, MA 02115, United States; University of California at San Francisco, San Francisco, CA 94143, United States; Division of General Internal Medicine, Brigham and Women's Hospital, Boston, MA 02115, United States; Department of Health Policy and Management, Harvard School of Public Health, Boston, MA 02115, United States; Division of Clinical Informatics and Digital Transformation, University of California at San Francisco, San Francisco, CA 94143, United States; Center for Physician Experience and Practice Excellence, Brigham and Women's Hospital, Boston, MA 02115, United States; University of California at San Francisco, San Francisco, CA 94143, United States; Philip R. Lee Institute for Health Policy Studies, University of California at San Francisco, San Francisco, CA 94158, United States; Division of General Internal Medicine, University of California at San Francisco, San Francisco, CA 94143, United States

**Keywords:** workforce, advanced practice providers, primary care, physicians, team-based care

## Abstract

More than 83 million people in the United States live in primary care shortage areas. As the US healthcare system faces a contracting primary care physician workforce, advanced practice providers are playing an increasingly important role in the delivery of primary care services. In parallel, public discourse regarding the differences in care delivery by advanced practice providers versus physicians has also expanded. In this commentary, we describe 3 main evidence gaps hindering optimal physician and advanced practice provider work organization in contemporary primary care delivery: (1) gaps in understanding the unique and overlapping competencies of each role group, (2) gaps in evaluating and defining optimal role delineation, and (3) gaps in payment models supporting effective collaboration. We subsequently present key needs in these 3 areas, including technology-based approaches to track physician and advanced practice provider competencies, increased empirical data on different clinical teaming structures, and exploration of novel models for primary care payment. We also note the need for an enhanced understanding of patient perspectives regarding primary care role types and teaming structures.

## Background

The US healthcare system faces a substantial crisis of primary care availability and access. More than 83 million people in the US live in primary care shortage areas.^1^ At the same time, more than 50% of active primary care physicians (PCPs) are over the age of 55^2^ and few graduates of internal medicine residency programs entering general medicine are choosing to enter outpatient primary care practice.^3^ Amidst multiple efforts to enhance the supply and capacity of the primary care physician workforce, advanced practice providers (APPs) are playing an increasingly important role in the delivery of primary care services. Thus, the role of APPs in internal medicine and especially in primary care, as well as how APPs collaborate with physicians have become questions of increasing importance.

Advanced practice providers—including physician assistants (PAs) and nurse practitioners (NPs)—represent more than half a million healthcare professionals across the United States, practicing across a variety of locations and specialties.^4^ While APPs must practice under the supervision of physicians in many parts of the United States, at present, 27 US states offer full practice authority to NPs, and 6 states have removed the requirement for PAs to be supervised by or collaborate with a physician.

As the prevalence of APPs in the United States has grown, public discourse regarding the benefits and drawbacks of care by APPs versus physicians has expanded, driven in part by professional societies representing both the groups. Alongside this discourse, questions have emerged related to APPs’ skills, quality of care delivery, and scope of practice; their roles within the healthcare team; and reimbursement for their work. While many studies document similar health outcomes among patients cared for by physicians versus APPs,^5-7^ this literature is limited by predominantly noncausal research designs and data limitations that fail to fully address selection bias.

These unanswered questions pose key roadblocks to optimizing contemporary primary care delivery. In March 2024, the (Physicians Foundation) Center for (Physician Experience and Practice Excellence) invited physicians, APPs, and subject matter experts to engage in a discussion of key unanswered questions related to physician and APP partnerships, with a goal of informing future research, operations, and policies.

Through this dialogue, additional review of the literature, and the integration of knowledge and perspectives from each of our research, policy, and operational roles, our authorship group identified 3 main evidence gaps hindering optimal physician and APP work organization in primary care: (1) gaps in understanding and defining the unique and overlapping competencies of each role group, (2) gaps in evaluating and defining optimal role delineation, and (3) gaps in payment models supporting effective collaboration. In this article, we describe key needs in these 3 areas, reflecting the perspectives of this authorship group. We also highlight the need for enhanced patient perspectives regarding care delivery by physicians vs APPs, and team-based vs single provider models of care.

## Understanding the competencies of each group

The quantitative differences in training between physicians and APPs are clear. While primary care physicians complete 4 years of medical school and 3 years of residency, including 12 000-16 000 patient care hours in training, the training of NPs and PAs is less time intensive. In general, NPs undertake master's or doctoral degrees in the advanced practice of nursing, which require 1.5-3 years of training and 500-1000 clinical hours beyond RN training, though direct entry programs allow for the training of NPs without prior nursing training or experience in 3 years. Nurse practitioners are trained in a clinical focus area such as family medicine, adult-geriatric care, pediatrics, or psychiatry/mental health. Meanwhile, PAs complete a master's degree delivered via in-person instruction, which includes 9-10 months of didactic training followed by 2000 h of clinical rotations. These include rotations in internal medicine, pediatrics, surgery and ob-gyn, and specialty electives, which can be completed in 2-3 years. While some PA residency opportunities are available for additional specialized training, these are not uniformly available or required before entering practice. Notably, the specifics and content of training can vary substantially across NP and PA programs.

While each profession has published its own expected set of competencies after graduation from a training program (eg, a physician assistant^8^ or nurse practitioner^9^ training program or medical residency for physicians^10,11^) and the concepts tested on each role type's certification exams are codified,^12-15^ it is less clear is how each specific training program translates to the proficiencies needed for different aspects of primary care practice. Perhaps more broadly, the competencies needed for primary care practice across role types have not been well defined, suggesting an opportunity to codify the skills, knowledge, and approaches needed for high-quality primary care delivery based on previously established standards. It is well recognized that the tasks of primary care are broad. They range from assessing acute medical issues to chronic disease management and include evaluating and developing plans for vague and chronic symptoms. While some of these tasks involve conforming to protocolized aspects of care (eg, following screening recommendations and some aspects of chronic disease management), some are less well defined and more cognitively intense, such as assessing the importance of and developing detailed, longitudinal diagnostic and treatment plans for vague and poorly characterized symptoms. Clearly defining the range of competencies needed to effectively practice modern primary care could help facilitate identification of where an individual practitioners’ specific strengths lie, the scope of practice they are prepared for, and which skills need to be further developed.

It should additionally be noted that while certain competencies may not be gained or perfected during training, they can potentially subsequently be developed or enhanced with years on the job. Although ongoing assessment and reporting of competencies would be ideal and valuable, this could be burdensome to individuals and organizations. In the future, automated electronic health record (EHR)-based reporting and artificial intelligence–based note assessment may provide an avenue through which to identify and categorize the range of patients and diagnoses seen by physicians vs APPs, and the actions taken and diagnostic reasoning displayed by each role group in specific clinical situations. As previously described by Rule et al.^16^ EHR activity logs have been used in multiple studies to analyze clinician workflows and the actions that clinicians undertake via the EHR. They have also been used to attribute patients to specific physicians in the primary care, in-patient, and emergency department settings.^17^ Meanwhile, artificial intelligence–based solutions, such as large language and machine learning models, have been shown to effectively summarize clinical note content^18^ and to annotate and summarize transcripts from Objective Structural Clinical Examinations,^19^ respectively. While still being optimized, these types of technology-based approaches may be a starting point for objective skills assessment and case tracking, complementing existing information about formal training and practical clinical experience.

## Evaluating and defining optimal role delineation

Both lack of clarity regarding each role group's competencies, as discussed above, and the relatively organic growth of the APP professions have generated unclear role delineation. For some APPs and physicians, lack of role clarity generates frustration—and even conflict—and can hinder optimal team functioning.^20^ This issue is particularly salient in primary care. In the original days of the patient-centered medical home, primary care leaders described an ideal physician–APP co-management partnership wherein primary care physicians saw new and more complex patients, while their APP partners engaged in more standardized chronic disease management and follow-up activities.^21^ Yet, current models of physician and APP practice in primary care vary widely. In some locations, primary care physicians and APPs share panels of patients. In others, NPs and physician assistants carry their own patient panels. In others, APPs see patients only for episodic care, see patients with specific chronic diseases, and/or to help with inbox management and patient communication. However, all combinations of these roles exist. In practice, role delineation is dictated by a combination of the training, experience, and confidence level of the APP, the needs of the practice, and the difficulty of adhering to clearly defined roles in the demanding and often unpredictable primary care environment.

Ultimately, more empirical data about optimal teaming structures are essential. There are strong empiric data about the value of continuity of care,^22^ supporting the importance of patients seeing a consistent practitioner or team over time. However, further studies are needed that rigorously evaluate the impact of different practice model arrangements (eg, physician alone, physician-APP stable dyad, consistent physician with rotating APP, NP/PA independent practice) on outcomes for diverse patients, ranging from young, healthy patients to patients with multiple comorbidities and large multidisciplinary care teams. It also would be helpful to evaluate how outcomes compare across the practice model arrangements based on the relative experience of each role group member, how patients perceive the differences between APP and physician encounters, and which activities are delivered best by the different roles. Ideally, these studies would also assess the satisfaction of practitioners working independently versus in teaming models. Generation of this evidence could inform primary care practice leaders and policymakers about how to design their teams to balance local workforce pressures, patient needs, and clinician satisfaction. We acknowledge, however, that these data will not be trivial to generate, given the need for strong quasi-experimental or randomized designs in order to draw accurate conclusions.

## Identifying payment models that support optimal collaboration

At present, Medicare payment for the work of APPs takes place in one of the 2 ways. Advanced practice providers can bill Medicare directly and receive 85% of the Medicare physician fee schedule reimbursement rate. Alternatively, if they meet specific conditions, APPs can bill Medicare indirectly “incident to” a supervising physician and the dyad then receive 100% of the fee schedule reimbursement. Recent estimates suggest that roughly 40% of APP-provided evaluation and management services are billed indirectly.^23^ While financially advantageous to providers, indirect billing renders APP-provided care nearly invisible in Medicare administrative claims data, constrains policymakers’ ability to evaluate the cost and quality of care delivered by different clinicians, and may increase out-of-pocket costs for Medicare beneficiaries.^24^ Even if APP-provided services were reimbursed at 100% of physician fee scheduled rates when billed directly, this would not solve well-documented discrepancies in reimbursement between primary care and specialist services. Additionally, reimbursing primary care using a fee schedule, as is done in Medicare and most private insurance arrangements, includes few, if any, financial incentives for optimal collaboration between physicians and APPs in team-based care, as traditional primary care reimbursement levels may not be enough to cover the costs of physician services, APP services, and other staff and infrastructure involved in high-quality, team-based primary care delivery.

Given demonstrated benefits of team-based care, novel models for primary care payment would ideally provide sufficient monetary support for multiple members of the primary care team and allow primary care practices to incorporate APPs as they see fit. In line with this approach, the recent NASEM primary care report called for reforming primary care pay with a focus on paying for value, in part to support such team-based delivery models.^25^ Previously studied models incorporating innovative payment approaches have demonstrated promise for enhancing team-based care delivery in general. For example, in the Center for Medicare and Medicaid Service's (CMS's) Comprehensive Primary Care Plus model, which provided care management fees to all practices and partial capitation fees to track 2 practices, participating primary care practices integrated new team members to provide care management, behavioral health, and pharmacist services. Additionally, participating practices saw a 11% increase in primary care practitioners, 67% of which was due to practices adding non-physician practitioners such as physician assistants and NPs, although exactly how these practitioners were integrated into practices was not defined.^26^ Similar efforts at expanding team roles have been reported as part of the initial evaluation for CMS's Primary Care First Model,^27^ and CMS's newer ACO Primary Care Flex Model also involves a prospective payment meant to resource and incentivize team-based primary care delivery among participating ACOs. Medicare Advantage plans, which have an incentive to optimize the care of their beneficiaries within a set payment, in theory also maintain the flexibility to incorporate such payment models, although evidence about teaming in Medicare Advantage plans is lacking. Ultimately, it remains to be seen the extent to which primary care-directed, value-focused payments will help primary care clinics optimize team design, and what role APPs will play on expanded teams. Additionally, further efforts are needed to enhance the appeal of these models to primary care practices, which are unlikely to change their team-based structures unless most of their patients are in value-based arrangements given sometimes competing incentives between fee-for-services and value-based care payment models. Indeed, one study projected that a shift to team-based and nonvisit-based care would only be financially beneficial to practices with >63% of payments deriving from capitated payments, thus limiting the current scalability of these approaches.^28^ Nevertheless, CMS has stated an interest in having 100% of Traditional Medicare beneficiaries and most Medicaid beneficiaries in accountable care relationships by 2030,^29^ potentially accelerating the spread of novel primary care payment approaches, and thus, team-based care delivery.

## The patient perspective

In addition to the questions regarding competency, organization of care, and payment highlighted above, better evidence is needed regarding how patients perceive care provided by physicians versus APPs and how they experience team-based models of care vs those centered on continuity with a single provider. Previous studies have described factors associated with patient preference for physicians versus APPs as primary care providers, including previous provider qualifications and patients’ previous healthcare experiences.^30^ They have also described how patients with multimorbidity in particular may value continuity of care with a single provider, even at the expense of sooner access to care.^31^ However, it would be beneficial for healthcare leaders to more thoroughly understand how patients experience and value care provided by physicians vs APPs, including how this impacts their engagement, trust, and satisfaction. Additionally, it will be important to more thoroughly characterize how patients perceive team-based care arrangements involving teams of physicians and APPs, and the specific patients who value collaborative arrangements versus those who value single provider continuity.

## Conclusion

As the United States continues to grapple with the PCP shortage, there is no doubt that APPs will remain an integral and growing part of the primary care workforce and in internal medicine more broadly. Ensuring their successful integration into this workforce may require concerted efforts to clarify key questions related to the skills of different members of these teams, how we evaluate and define optimal role delineation, how we pay for the care models that facilitate optimal practice, and the experience of patients.

## Supplementary Material

qxaf006_Supplementary_Data

## Notes

B.L.'s time was funded by P01AG032952 from the National Institutes of Ageing. B.L. reports consulting fees from Freedman Healthcare Consulting, the American Board of Internal Medicine, RTI, Inc., and UpTodate. He reports honoraria from Upstream and CVS and payment for expert testimony from Fowler, White, and Bernett. D.W.B.'s time was supported by the Physicians Foundation. He reports grants from EarlySense, SCI Negev, and IBM Watson Health, equity from CLEW, MDClone, and Guided Clinical Solutions, and personal fees and equity from AESOP and Feel Better Inc. H.N.'s time was supported by Arnold Ventures and the Agency for Healthcare Research and Quality (5R03HS028490). L.R.'s time was supported by the Physicians Foundation. She reports grants from Feel Better, Inc. and the American Medical Association, honoraria from Phreesia, Inc., serving on the AI Advisory Board for Augmedix, Inc., and travel support from the American College of Physicians and FeelBetter, Inc. S.E.L. reports grants from AHRQ and PCORI, consulting fees from N1X!0, honoraria from the Harvard School of Public Health and Harvard Medical School, and support for travel from the American Board of Internal Medicine. J.S. reports consulting fees from the Palo Alto Veterans Affairs Healthcare System, UnitedHealth Group, and Columbia University, honoraria from the Center for the Future of Arizona and the American Medical Directors Association, and payment for expert testimony from the Planned Parenthood Foundation. She additionally reports support for meeting attendance from America's Physician Groups, the National Academy for State Health Policy, the California State University Chancellor's Office, and the California Organization of Associate Degree Nursing & California Association of Colleges of Nursing.
