# Supplementary material for: An unclear partnership: key questions about physician and advanced practice provider collaboration in primary care
Source: Health Aff Sch. 2025 Jan 17;3(2):qxaf006. doi: 10.1093/haschl/qxaf006 (PMC11842302; doi:10.1093/haschl/qxaf006)
Supplement: qxaf006_Supplementary_Data [file qxaf006_supplementary_data.zip › coi_disclosure_JoanneSpetz.docx]

| ICMJE DISCLOSURE FORM | |
| --- | --- |
| **Date:** | 10/21/2024 |
| **Your Name:** | Joanne Spetz |
| **Manuscript Title:** | **An Unclear Partnership: Key Questions about Physician and Advanced Practice Provider Collaboration in Primary Care** |
| **Manuscript Number (if known):** | 2024-01359 |
| In the interest of transparency, we ask you to disclose all relationships/activities/interests listed below that are related to the content of your manuscript. “Related” means any relation with for-profit or not-for-profit third parties whose interests may be affected by the content of the manuscript. Disclosure represents a commitment to transparency and does not necessarily indicate a bias. If you are in doubt about whether to list a relationship/activity/interest, it is preferable that you do so.  The author’s relationships/activities/interests should be defined broadly. For example, if your manuscript pertains to the epidemiology of hypertension, you should declare all relationships with manufacturers of antihypertensive medication, even if that medication is not mentioned in the manuscript.  In item #1 below, report all support for the work reported in this manuscript without time limit. For all other items, the time frame for disclosure is the past 36 months. | |

|  | | | **Name all entities with whom you have this relationship or indicate none (add rows as needed)** | **Specifications/Comments (e.g., if payments were made to you or to your institution)** |
| --- | --- | --- | --- | --- |
| **Time frame: Since the initial planning of the work** | | | | |
| **1** | All support for the present manuscript (e.g., funding, provision of study materials, medical writing, article processing charges, etc.)  **No time limit for this item.** | | \|  \| **None** \| \| --- \| --- \|  \|  \|  \| \| --- \| --- \| \|  \|  \| \|  \| Click the tab key to add additional rows. \| | |
| **Time frame: past 36 months** | | | | |
| **2** | | Grants or contracts from any entity (if not indicated in item #1 above). | \|  \| **None** \| \| --- \| --- \|  \| US Health Resources and Services Administration \| Paid to institution \| \| --- \| --- \| \| Massachusetts Health Policy Commission \| Paid to institution \| \| National Institutes of Health \| Paid to institution \| \| University of California, Berkeley \| Paid to institution (subaward; primary grant from Agency for Healthcare Research and Quality) \| \| California Department of Aging \| Paid to institution \| \| California Department of Health Care Access and Information \| Paid to institution \| \| The California Endowment \| Paid to institution \| \| California Board of Registered Nursing \| Paid to institution \| \| University of Michigan \| Paid to institution (subaward; primary grant NIH) \| \| California Department of Justice \| Paid to institution \| \| Northern California Institute for Research & Education \| Paid to institution (subaward; primary grant NIH) \| \| California Bureau of Cannabis Control \| Paid to institution \| \| Oregon Health Authority \| Paid to institution \| \| Sacramento County Department of Health Services \| Paid to institution \| \| UC Tobacco-Related Disease Research Program \| Paid to institution \| \| CA Health & Human Services Agency \| Paid to institution \| \| California Health Care Foundation \| Paid to institution \| \|  \|  \| \|  \|  \| | |
| **3** | | Royalties or licenses | \|  \| **None** \| \| --- \| --- \|  \|  \|  \| \| --- \| --- \| \|  \|  \| \|  \|  \| | |
| **4** | | Consulting fees | \|  \| **None** \| \| --- \| --- \|  \| Palo Alto Veterans Affairs Health Care System \| Paid to me for consulting \| \| --- \| --- \| \| UnitedHealth Group \| Honorarium for Advisory Board membership \| \| Columbia University \| Research consulting \| \|  \|  \| | |
| **5** | | Payment or honoraria for lectures, presentations, speakers bureaus, manuscript writing or educational events | \|  \| **None** \| \| --- \| --- \|  \| Center for the Future of Arizona \| Speaker honorarium, travel expenses \| \| --- \| --- \| \| American Medical Directors Association \| Speaker honorarium \| \|  \|  \| | |
| **6** | | Payment for expert testimony | \|  \| **None** \| \| --- \| --- \|  \| Planned Parenthood Federation \| Paid to me for expert witness consulting \| \| --- \| --- \| \|  \|  \| \|  \|  \| | |
| **7** | | Support for attending meetings and/or travel | \|  \| **None** \| \| --- \| --- \|  \| America’s Physician Groups \| Travel expenses for conferences, 2023 & 2024 \| \| --- \| --- \| \| National Academy for State Health Policy \| Travel expenses for conference \| \| California State University Chancellor’s Office \| Travel expenses for conference \| \| California Organization of Associate Degree Nursing & California Association of Colleges of Nursing \| Travel expenses for conference \| | |
| **8** | | Patents planned, issued or pending | \|  \| **None** \| \| --- \| --- \|  \|  \|  \| \| --- \| --- \| \|  \|  \| \|  \|  \| | |
| **9** | | Participation on a Data Safety Monitoring Board or Advisory Board | \|  \| **None** \| \| --- \| --- \|  \| National Health and Aging Trends Survey Data Monitoring Committee \| Member (pending) \| \| --- \| --- \| \| California Nursing Education and Workforce Advisory Committee \| Member \| \|  \|  \| | |
| **10** | | Leadership or fiduciary role in other board, society, committee or advocacy group, paid or unpaid | \|  \| **None** \| \| --- \| --- \|  \|  \|  \| \| --- \| --- \| \|  \|  \| \|  \|  \| | |
| **11** | | Stock or stock options | \|  \| **None** \| \| --- \| --- \|  \|  \|  \| \| --- \| --- \| \|  \|  \| \|  \|  \| | |
| **12** | | Receipt of equipment, materials, drugs, medical writing, gifts or other services | \|  \| **None** \| \| --- \| --- \|  \|  \|  \| \| --- \| --- \| \|  \|  \| \|  \|  \| | |
| **13** | | Other financial or non-financial interests | \|  \| **None** \| \| --- \| --- \|  \|  \|  \| \| --- \| --- \| \|  \|  \| \|  \|  \| | |
|  | |  |  | |
| **Please place an “X” next to the following statement to indicate your agreement:** | | | | |
|  | | I certify that I have answered every question and have not altered the wording of any of the questions on this form. | | |
